# Treatment of Bullous Systemic Lupus Erythematosus

**DOI:** 10.1155/2015/167064

**Published:** 2015-05-18

**Authors:** Lihua Duan, Liying Chen, Shan Zhong, Ying Wang, Yan Huang, Yan He, Jie Chen, Guixiu Shi

**Affiliations:** ^1^Department of Rheumatology and Clinical Immunology, The First Hospital of Xiamen University, Xiamen 361003, China; ^2^Department of Pathology, The First Hospital of Xiamen University, Xiamen 361003, China; ^3^Department of Immunology, College of Medicine, Xiamen University, Xiamen 361102, China

## Abstract

Bullous systemic lupus erythematosus (BSLE) is an autoantibody-mediated vesiculobullous disease in patients with SLE. Autoimmunity in BSLE is characterized by the presence of circulating anti-type VII collagen antibodies. BSLE patients often present with multiple, tense, clear fluid-filled vesicles and bullae overlying erythematous edematous plaques. Skin biopsy from BSLE patients shows subepidermal bullae with numerous neutrophils and only occasional eosinophils. Furthermore, immunofluorescence examination showed linear deposition of lgG, lgA, C3, and C1q along the basement membrane zone. BSLE patients with corticosteroids treatment constantly do not receive a marked improvement, while dapsone generally dramatically improved the skin condition. Recently, it has been reported that quite a few cases of BSLE were successfully treated with other immune suppressive drugs. Therefore, a comprehensive review of the treatment of BSLE would be beneficial to cure the disease.

## 1. Introduction

Bullous systemic lupus erythematosus (BSLE) is a subepidermal blistering disease that occurs in a subset of patients with systemic lupus erythematosus (SLE) [1, 2]. Cutaneous lesions are reported during the course of SLE in 76% of patients; however, it has been reported that BSLE is very rare and occurs in less than 1% of patients with SLE [3–5]. Clinically, in addition to the features of SLE, the BSLE patients especially present with a rapid, widespread development of tense fluid-filled vesicles and bullae. Moreover, this blistering disease may vary from a small group of vesicles to large tense blisters with urticarial eruptions, erosions, itching, and crustations. Histologically, BSLE is characterized by a subepidermal blister, with a predominantly neutrophilic dermal infiltrate and only occasional eosinophils. Furthermore, immunofluorescence examination showed linear deposition of lgG, lgA, C3, and C1q along the basement membrane zone [5, 6]. Because of the particularly clinical and histological presentation of BSLE, Camisa and Sharma proposed diagnostic criteria for BSLE; these include a diagnosis of SLE based on the following criteria of the ACR; vesicles and bullae mainly located on sun-exposed areas; the histopathology is characterised by subepidermal bullae with microabscesses of neutrophils in the dermal papillae, similar to those found in dermatitis herpetiformis and deposition of IgG, IgM, or both and often IgA in the basement membrane zone [7].

Although BLSE may exhibit any of the symptoms associated with SLE, the onset and course of blistering eruption do not necessarily parallel the activity of the systemic involvement [8]. Furthermore, the therapeutic options for SLE are not usually fit for BSLE [9, 10]. In some cases, the eruption flared after systemic corticosteroid administration for SLE [11, 12]. However, most of the patients have a striking therapeutic response to dapsone [13–16]. A response may be seen with very small doses of dapsone [1]. In the case of the present paper, we report a significantly and clinically meaningful improvement of BSLE following dapsone administration. Other drugs such as cyclophosphamide, azathioprine, and mycophenolate mofetil and biologic drugs may also be effective for BSLE treatment [17]. In the part of literature review, we provide a review of all the available treatment options for BSLE.

## 2. Literature Review

**Figure 1:**
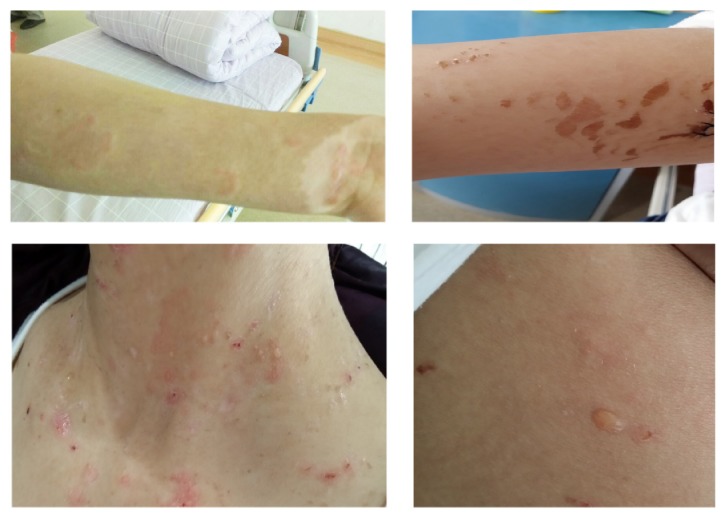
Gross view of the skin lesion. Presence of tense vesicles (marked with an arrow) filled with clear fluid on the arm, neck, and back.

**Figure 2:**
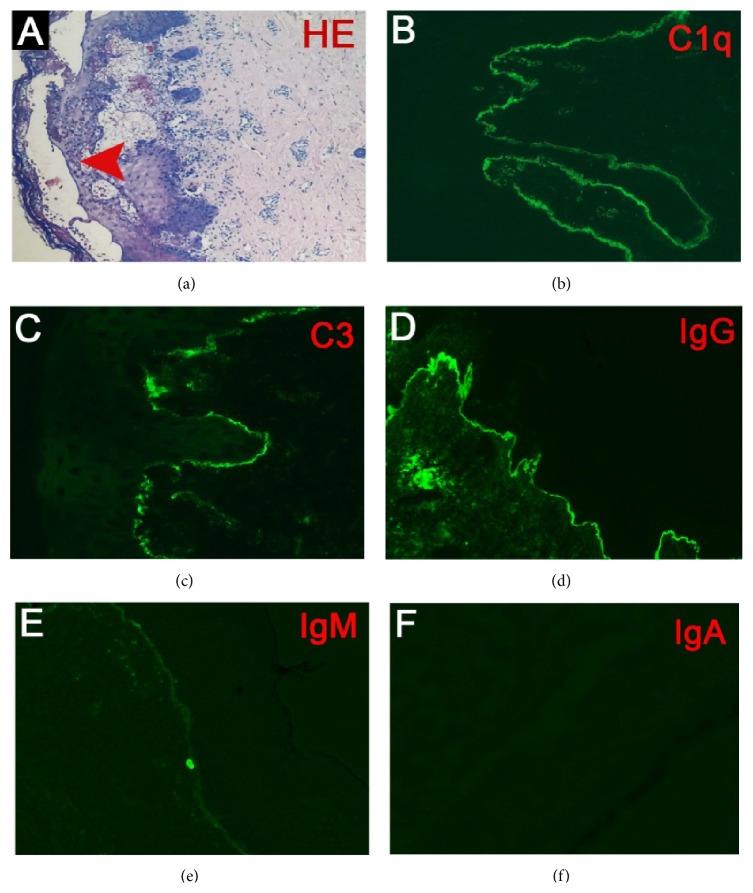
Histopathology of the skin lesion. (a) Histopathologic examination of the skin biopsy specimen showed a subepidermal blister (arrow indicated) with abundant neutrophils infiltration and only occasional eosinophils (H&E stain, 100x). (b) Direct immunofluorescence examination showed linear, granular deposition of C1q (b), C3 (c), and IgG (d) at the dermoepidermal junction (400x); less IgM and IgA were detected.

Steroids and antimalarials are the standard treatments for the cutaneous manifestations of SLE. In unresponsive patients, azathioprine and high dose or pulse steroids, cyclosporin, and pulse cyclophosphamide are the most commonly used alternative therapies [18–20]. Dapsone is less used in the control of the SLE rash but has a dramatic improvement in the eruption of BSLE patients [15, 16]. A relatively low dose has also been shown to be an efficacious response. We also found that a 22-year-old woman with BSLE had multiple tense vesiculobullous lesions on the face, trunk, and limb (Figure 1). A biopsy from the upper limb showed a subepidermal blister with a predominantly neutrophilic dermal infiltrate and only occasional eosinophils (Figure 2(a)). Immunofluorescence showed a granular band of C1q, C3, and IgG at the basement membrane; less IgA and IgM were observed (Figures 2(b)–2(f)). The skin condition showed no response in the methylprednisolone, while a considerable improvement after dapsone administration was observed. Regarding the special clinical feature and the discriminative therapies from the SLE treatments, we review all the available treatment for BSLE.

### 2.1. Dapsone

Dapsone is a sulfone that has played a critical role in the eradication of leprosy [21]. Besides, a number of cutaneous eruptions are effectively controlled by dapsone [22]. Due to these eruptions that are largely characterized by the presence of cutaneous neutrophilic dermal infiltrate [22], such as dermatitis herpetiformis and the inflammatory variant of epidermolysis bullosa acquisita, the mechanism of its anti-inflammatory action mainly relies upon its inhibition of the functions of polymorphonuclearleukocytes and of complement activation via the alternative pathway that has been postulated [15, 23]. Although a new or recurrent rash was considered a factor of SLE disease activity index, the eruption of BSLE was not constantly associated with a flare of SLE [8]. Consequently, because of being unparallel with the disease activity, the eruption of BSLE patients is often unresponsive to corticosteroid therapy. Due to the striking histologic resemblance to dermatitis herpetiformis, the patients who were treated with dapsone (2 mg/kg/day) usually obtained a dramatic improvement in the eruption. Patients tend to have an efficacious response with cessation of new blister formation in 1-2 days and healing of existing lesions within several days. A relatively low dose (25–50 mg) has also been shown to have a response [1, 15, 24]. Interestingly, improvement of the eruption did not correlate with amelioration of the systemic manifestations [25]. The dramatic response to dapsone therapy demonstrated that dapsone is useful in treating bullous lesions of SLE [5, 15, 16, 26]. Notably, the blistering eruption was not improved by dapsone in some cases, and even worsening has been noted after its administration. It has been reported that patients with BSLE, who initially presented with lesions clinically resembling erythema multiform, experienced exacerbation of their disease with dapsone [27–29]. Furthermore, dapsone has been assigned to pregnancy category C; BSLE patients with pregnancy might not be fit for administration of this drug [30]. Hemolysis and hepatic and renal toxicity usually accompany administration of the drug in a dose related fashion [31–34]; therefore its clinic use was confined and a careful monitoring of its toxicity is required.

### 2.2. Corticosteroids

Corticosteroids are usually required to improve clinical symptoms and laboratory abnormalities and are still a mainstay for inducing remission in SLE patients [18, 20, 35]. Topical corticosteroids may be helpful in the treatment of cutaneous SLE. Unexpectedly, many bullous SLE patients tend to be unresponsive to systemic corticosteroid therapy that has been described [36]. Furthermore, in some cases of SLE patients, the eruption flared a few days after systemic corticosteroid administration [11, 12]. Interestingly, some patients responded effectively to corticosteroids, although they required relatively high doses [37]. A patient with SLE who presented with vesiculobullous lesions during the third trimester of pregnancy has been presented. A skin biopsy of this patient was performed, and it showed significant necrosis of keratinocytes in the epidermis and granular, dense, and continuous deposits of moderate IgG positivity in the basement membrane zone. Dapsone has been assigned to pregnancy category C. The pregnant woman treated with high-dose corticosteroids obtained a satisfactory response [30]. As the dapsone administration often causes hepatic and renal toxicity [32, 33, 38–40], prednisone alone or in combination with low doses dapsone might be the treatment of choice for these BSLE patients. These observations demonstrated that corticosteroids may act as an alternative treatment for BSLE when patients are unresponsive or unfit for other drugs [37].

### 2.3. Rituximab

Biologic agents, such as infliximab, rituximab, and anakinra, have emerged as effective therapies for treating a wide spectrum of diseases which includes various rheumatic, gastrointestinal, and cutaneous diseases [41–44]. The involvement of all of the key components (especially cytokines and immune cells) of the immune system in the pathogenesis of SLE offers many potential targets for therapeutic management of this disease. B cells, a critical immune cell, which can act as antigen-presenting cells, differentiate into plasma cell to produce pathogenic autoantibodies and secrete various cytokines and chemokines in the immune response [45, 46]. These functions of the B cell support the fact that it plays an important role in the development of pathogenesis of SLE. Therefore, use of B cell depletion therapy in SLE has emerged as a novel and promising therapeutic alternative for SLE patients [47–49]. Rituximab which is a chimeric monoclonal antibody that reacts with CD20, an antigen that is present on immature, naive, and memory B cells but not on mature plasma cells, has been approved in the treatment of SLE [50]. Up to now, there is only a case report about rituximab in BSLE. The patient was treated with hydroxychloroquine (HCQ) twice daily, mycophenolate mofetil 1000 mg/d, and varying doses of corticosteroids, while the eruption was not improved. Dapsone and azathioprine were added but had to be stopped because of elevated liver enzymes and leukopenia. Mycophenolate mofetil was increased to 2000 mg/d, but her skin disease remained active. Then the patient was treated with intravenous infusions of rituximab. The skin lesions improved within 10 days after the first dose and cleared by day 15 after the second dose. Furthermore, prednisone was successfully tapered, and the patient has remained free of recurrence of cutaneous and oral blistering lesions [34]. The results of this case suggest a potential role for treatment of refractory BSLE with rituximab.

### 2.4. MTX

Methotrexate (MTX) has been widely proved to be an effective agent in control of the rheumatoid arthritis [51]. It has proved that MTX was beneficial in sporadic cases of SCLE refractory to therapy with conventional therapy [52–54], such as antimalarials and corticosteroids. Furthermore, in a randomized and double-blind trial in 41 patients with SLE, MTX reveals a role for controlling the skin lesions in 75% of cases, with a mean reduction of prednisone dose of 44% [55]. These reports demonstrated that MTX could represent a valid therapeutic option in controlling the cutaneous SLE and in sparing the steroid dose. However, BSLE is a subepidermal blistering disease that occurs in a subset of patients with systemic lupus erythematosus (SLE). Cutaneous lesions of BSLE are reported during the course of SLE in less than 1% of patients, while lesions are not in line with the disease activity [9]. Unexpectedly, BSLE is often unresponsive to antimalarials and corticosteroids [56]. In a recent report, a rapid and full resolution of cutaneous lesions was obtained with methotrexate alone. A case of 40-year-old female with systemic lupus erythematosus (SLE) developed a severe bullous eruption on sun-exposed areas, while the previous manifestations of the disease were quiescent. In consideration of prior intolerance to many drugs, methotrexate was administrated. The drug administration was followed by a rapid and full resolution of cutaneous lesions. Therefore, MTX might be an alternative therapeutic choice to dapsone [57].

### 2.5. Other Therapies

HCQ is a commonly used drug for controlling the cutaneous lesions and the disease activity of the SLE [58, 59], while it does not act effectively in the eruption of BSLE [10]. The conventional treatment for SLE only revealed modest improvement in steroids and antimalarials [9]. Although cyclophosphamide has been shown to produce moderate improvement of skin lesions of SLE, the beneficial role in BSLE has not been demonstrated [60]. Mycophenolate mofetil (MMF) has been widely used for suppressing the lupus activity, while it also was not valid for BSLE, even at a high dose [10, 37]. In cases nonresponsive to dapsone, the eruption has been controlled by prednisone or with combination therapy of prednisone and azathioprine [8].

## 3. Conclusions

In the treatment of bullous SLE, dapsone is the effective basic therapy, and it often induces a dramatic response. In some cases, where an adequate response is not achieved with dapsone or the SLE disease activity index is high, other immunosuppressants, such as prednisolone, methotrexate, and azathioprine, can be used for controlling the eruption and suppressing the systemic symptoms. Moreover, in some special case, where dapsone administration or other chemical drugs (MTX, azathioprine, etc.) induce serious side effects, biologic agents might be an alternative choice for BSLE.
